# Robot Assisted Stereotactic Laser Ablation for a Radiosurgery Resistant Hypothalamic Hamartoma

**DOI:** 10.7759/cureus.581

**Published:** 2016-04-21

**Authors:** Nicholas Brandmeir, Vinita Acharya, Michael Sather

**Affiliations:** 1 Department of Neurosurgery, Penn State Milton S Hershey Medical Center; 2 Department of Neurology, Penn State Milton S Hershey Medical Center

**Keywords:** hypothalamic hamartoma, gelastic seizure, laser, frameless, stereotactic, robotic surgery, rosa

## Abstract

Hypothalamic hamartomas (HH) are benign tumors that can cause significant morbidity in adults as a cause of epilepsy, particularly gelastic seizures. Open and endoscopic resections of HH offer good seizure control but have high rates of morbidity and are technically challenging. Stereotactic radiosurgery has been an alternative treatment; however, it results in comparably poor seizure control. Recently, in children, stereotactic laser ablation has shown promise as a surgical technique that can combine the best features of both of these approaches for the treatment of HH. Here we present the first reported use of a frameless robot-assisted stereotactic system to treat an HH. The patient had failed two previous Gamma Knife radiosurgery treatments. Post-procedure he had a stable, but unintentional weight loss of 20 kg and a transient episode of hemiparesis the night of the operation. At six months postoperatively the patient remained seizure free. Stereotactic laser ablation may represent a new standard in the treatment of HH in adults, especially in those who have failed radiosurgery. Further study is warranted in this population to determine efficacy and safety profiles.

## Introduction

Hypothalamic hamartomas (HH) are benign growths of glioneuronal tissue that are centered on the tuber cinereum. They are commonly associated with medically refractory epilepsy, developmental delay, and precocious puberty [1]. The most common seizure semiology in patients with HH is gelastic seizures (GS). Gelastic seizures are characterized by uncontrolled bursts of laughter with maintained consciousness and without the usual elevated mood [1]. The presence of GS can also lead to a kindling effect leading to more complicated seizure semiology as well as development of neuropsychological deficits [2]. For these reasons control of the GS is imperative.

It has been well described that surgical resection of HH can make patients free of their GS [3]. Despite this, there is no agreed upon optimal surgical approach to the HH and many previously described techniques have low rates of seizure freedom (~50%) and relatively high rates of complications (~25%) [4-6]. 

Stereotactic Laser Ablation (SLA) was introduced as a treatment for HH because of the shortcomings of previous treatment methods. Stereotactic Laser Ablation consists of using a stereotactically placed laser with a diffusing tip to cause a thermal injury while using real-time magnetic resonance imaging thermography to monitor temperature and ensure the lesion is restricted to the appropriate structures [7]. In a series of 14 pediatric patients, Wilfong and Curry achieved a seizure freedom rate of 80% without clinical complications [8].

Previous reports of SLA for HH have relied on the use of stereotactic frames to place the laser device accurately [2,8]. Frameless systems have been developed that offer several advantages over traditional frames including more convenient pre-planning, faster operating room times, greater patient comfort and equivalent accuracy [9,10]. Our report focuses on the first reported use of robot-assisted SLA for HH in the literature.

## Technical report

**Figure 1:**
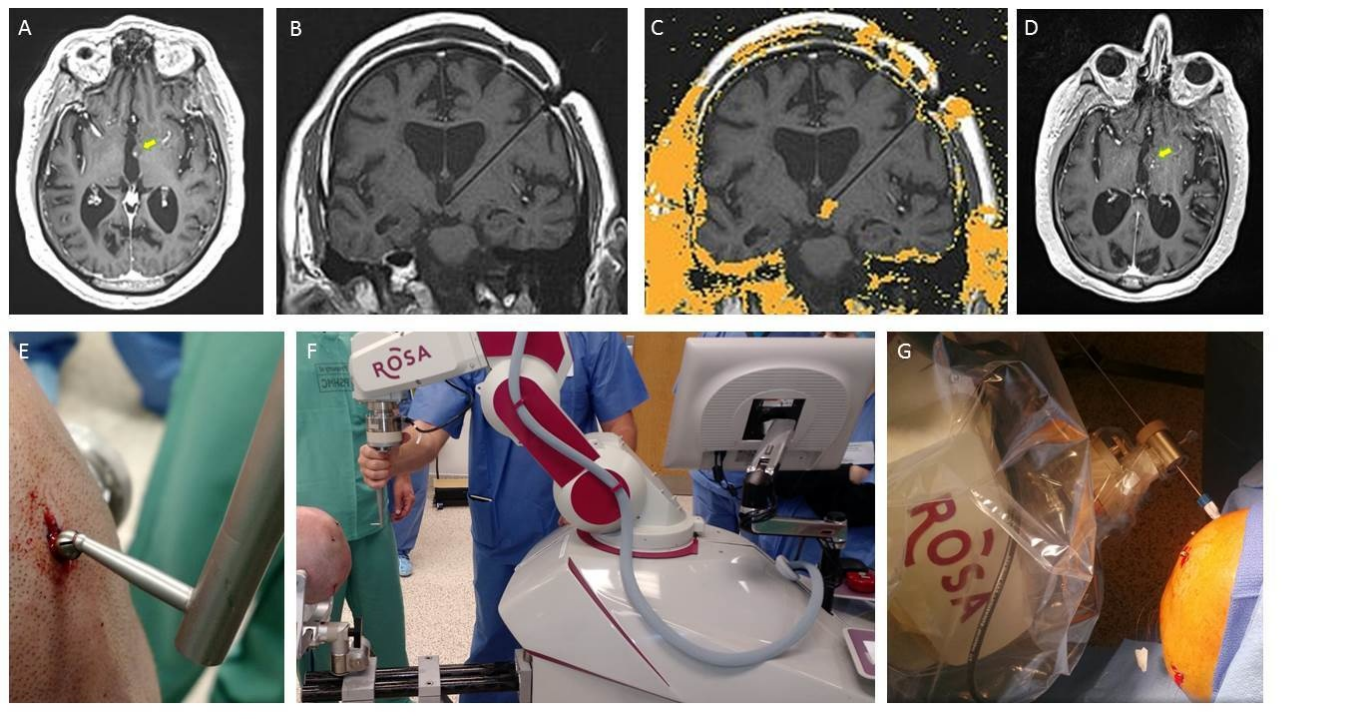
Imaging and operative details for a robot-assisted, frameless stereotactic laser ablation for an adult hypothalamic hamartoma A: Axial T1 post-contrast MRI demonstrating the left HH (yellow arrow); B: Coronal T1 MRI demonstrating accurate stereotactic placement of Visualase catheter; C: MRI thermography derived area of predicted lesion; D: Axial T1-post-contrast image on postoperative-day 1 demonstrating the lesion confined to the HH (yellow arrow); E: Demonstration of the ROSA registration arm interfacing with the Medtronic bone fiducial; F: Operative positioning of the ROSA robot and patient for registration and eventual Visualase catheter placement; G: Photograph demonstrating placement of the Visualase skull bolt with the ROSA robot in poly-axial guidance mode.

The patient is a 63-year-old man with a history of GS since the age of three. Imaging had consistently demonstrated a 1 cm non-enhancing mass on the wall of the left hypothalamus (Figure 1). All treating physicians involved in the case felt that this mass represented an HH. As a child he also experienced generalized tonic-clonic seizures, but, as an adult, only auras of “riding an elevator.” Seven years and again one year prior to presenting again for surgical management, the patient had undergone Gamma Knife Radiosurgery (GKS) for his HH. The first GKS treatment consisted of one 8 mm collimated shot treated to 17 Gy to 50% isodose line. The second GKS treatment consisted of another 8 mm collimated shot delivering 17 Gy to the 50% isodose line. He did experience some temporary decrease in GS frequency after each procedure, but his GS returned to their usual rate within two months each time. After being made aware of the option of SLA and the associated risks and benefits, the patient wished to proceed with surgery. At that time informed consent for the procedure and the potential preparation of academic materials related to the procedure were obtained.

Prior to the day of surgery, an outpatient high-definition non-contrast computed tomography (CT) scan of the brain was obtained as well as a high-definition double-contrasted magnetic resonance imaging (MRI) of the brain. These were used to pre-plan a stereotactic trajectory to the HH through the left frontal lobe with the ROSA™ planning software (MedTech Surgical Inc., Newark, NJ). On the day of surgery, five bone fiducial markers (Medtronic Inc., Minneapolis, MN) were placed in the same-day perioperative unit and a computerized tomography scan was obtained. This was then merged with the pre-designed plan. After anesthesia induction, the patient’s head was immobilized in a Mayfield head clamp and this was attached to the ROSA robot. The ROSA was then registered to the bone fiducials and anatomic accuracy was verified by navigating to verifiable anatomic landmarks (Figure 1).

After registration, the ROSA was used to navigate to the pre-planned trajectory, and in poly-axial mode a 3.2 mm twist-drill hole was made in the skull and the Visualase skull bolt (Medtronic Inc, Minneapolis, MN) was screwed into position along the appropriate stereotactic trajectory. After this, the distance to the target was measured with the ROSA software and then the cooling sheath was inserted to the appropriate depth and secured with the bolt. Finally, the laser was inserted into the sheath and the patient was then removed from head fixation and transferred to the diagnostic MRI under general anesthesia. 

In the MRI, accurate targeting of the laser catheter was confirmed with placement of the catheter into the lesion (Figure 1) and then the ablation was carried out in two separate burns. Burn one consisted of an intensity of 4 watts for 84 seconds then at 4.5 watts for 180 seconds. The laser was then inserted 0.5 cm deeper along the trajectory to ensure complete ablation of the lesion and another ablation was undertaken. Intensity settings for this ablation were 3 watts for 43 seconds and 5.5 watts for 192 seconds. The estimated treatment damage map from the thermography data overlapped nicely with the post-contrast magnetic resonance images and the ablation of the HH was considered complete (Figure 1). As described in Rolston and Chang, areas near the ablation can be marked to ensure they are not damaged during the ablation [2]. We identified the optic tract, mammillary body and lateral hypothalamus for protection during the procedure.

After the procedure the patient was extubated without difficulty. He had transient right-sided weakness on the night of the operation that resolved with one dose of 10 mg of dexamethasone and never returned. He was neurologically intact, ambulating, tolerating a diet the next morning and was discharged home. Six months after the procedure, the patient remains seizure free. Interestingly the patient, who is obese (141 kg preoperatively) did report an approximate 20 kg unintentional weight loss after surgery. This occurred over the first two postoperative months and stabilized. By six months, his appetite had returned to normal but the weight loss was stable. No other hormonal or memory side-effects were detected. Three months postoperatively he did develop a deep venous thrombosis of the leg that required hospitalization and eventual rehabilitation. It was unclear if this was a complication related to the procedure or more related to his medical co-morbidities. At his six-month follow-up visit, the patient was neurologically intact, seizure free, down to two anti-epileptic drugs from three, and had an improved mentation, alertness, and speech fluency compared to his preoperative function.

## Discussion

This is the first report of the use of the ROSA stereotactic robot for the SLA of an HH. The success of this case demonstrates that this frameless robotic stereotactic system is accurate, as confirmed by the intraoperative MRI and postoperative changes in the HH on T1-MRI, for the lesioning of deep structures in a manner that is safe and effective. It may allow this procedure to be carried out more quickly and with less use of hospital resources. Also, replacing the stereotactic frame with simple bone fiducials may make the procedure easier to tolerate for awake patients, decreasing barriers to patient utilization and potentially increasing access to surgery for epilepsy patients. 

We also confirm the findings reported in the pediatric population and young adults [2,8] that SLA is potentially effective in controlling GS secondary to HH and is useful as a salvage procedure after patients have failed GKS. 

The patient did have brief hemiparesis after waking from the procedure, but this resolved within a few hours after a single dose of dexamethasone. To our knowledge, this is the first report of SLA in an HH to demonstrate a hemiparesis that was transient after a successful ablation. Further, the weight loss experienced by the patient, while certainly within the realm of expected side effects of HH ablation, is also a first-time report. This will have to be followed, as surgical treatments of the hypothalamus are an active area of clinical investigation in stereotactic and functional neurosurgery for the treatment of obesity [11]. To date, no lesioning techniques have been described for this purpose, but SLA may prove to be an optimal modality.

In conclusion, frameless robot-assisted SLA may be an effective and safe method for the treatment of HH in adults with GS who have failed stereotactic radiosurgery. Considering the potential decrease in morbidity when compared to endoscopic resection [5] and the relatively high efficacy as demonstrated in children [8], SLA may be the treatment of choice in adults with HH. This application will require further research. This paper also demonstrates the potential role of SLA for patients with HH who have failed GKS and who do not wish to undergo resection. Finally, this report highlights some complications that may be unique to adults or to adults who have had previous radiosurgery, specifically a transient acute, postoperative hemiparesis and unintentional weight loss. Fortunately, in the case of our patient, side effects were either temporary or clinically silent, but further study of this technique in this patient population will be necessary to determine its full side effect profile.

## Conclusions

Robot-assisted frameless SLA for HH can be carried out safely and may provide excellent seizure control even in patients for whom traditional therapies have failed. Further studies are needed to determine its side effects and risks.

## References

[1] Striano S, Striano P, Coppola A, Romanelli P (2009). The syndrome gelastic seizures – hypothalamic hamartoma: severe, potentially reversible encephalopathy. Epilepsia.

[2] Rolston JD, Chang EF (2016). Stereotactic laser ablation for hypothalamic hamartoma. Neurosurg Clin N Am.

[3] Berkovic SF, Arzimanoglou A, Kuzniecky R, Harvey AS, Palmini A, Andermann F (2003). Hypothalamic hamartoma and seizures: a treatable epileptic encephalopathy. Epilepsia.

[4] Mathieu D, Deacon C, Pinard C-A, Kenny B, Duval J (2010). Gamma Knife surgery for hypothalamic hamartomas causing refractory epilepsy: preliminary results from a prospective observational study. J Neurosurg.

[5] Rekate HL, Feiz-erfan I, Ng Y, Gonzalez LF, Kerrigan JF (2006). Endoscopic surgery for hypothalamic hamartomas causing medically refractory gelastic epilepsy. Childs Nerv Syst.

[6] Abla A, Shetter A, Chang S, Wait S, Brachman D, Ng Y, Rekate H, Kerrigan J (2010). Gamma knife surgery for hypothalamic hamartomas and epilepsy: patient selection and outcomes. J Neurosurg.

[7] McNichols RJ, Gowda A, Kangasniemi M, Bankson JA, Price RE, Hazle JD (2004). MR thermometry-based feedback control of laser interstitial thermal therapy at 980 nm. Lasers Surg Med.

[8] Wilfong AA, Curry DJ (2013). Hypothalamic hamartomas: optimal approach to clinical evaluation and diagnosis. Epilepsia.

[9] Gonzalez-Martinez J, Vadera S, Mullin J, Enatsu R, Alexopoulos A, Patwardhan R, Bingaman W, Najm I (2014). Robot-assisted stereotactic laser ablation in medically intractable epilepsy: operative technique. Neurosurgery.

[10] Vadera S, Chan A, Lo T, Gill A, Morenkova A, Phielipp N, Hermanowicz N, Hsu F (2015). Frameless stereotactic robot-assisted subthalamic nucleus deep brain stimulation: case report. World Neurosurg.

[11] Ho AL, Sussman ES, Zhang M, Pendharkar A V, Azagury DE, Bohon C, Halpern CH (2015). Deep brain stimulation for obesity. Cureus.

